# Patterns of sexual mixing with respect to social, health and sexual characteristics among heterosexual couples in England: analyses of probability sample survey data

**DOI:** 10.1017/S0950268814002155

**Published:** 2014-08-28

**Authors:** P. PRAH, A. J. COPAS, C. H. MERCER, A. NARDONE, A. M. JOHNSON

**Affiliations:** 1 University College London, Research Department of Infection & Population Health, London, UK; 2 Public Health England, HIV & STI Department, London, UK

**Keywords:** Assortative, health survey, sexual mixing, STI transmission

## Abstract

Patterns of sexual mixing are major determinants of sexually transmitted infection (STI) transmission, in particular the extent to which high-risk populations mix with low-risk populations. However, patterns of mixing in the general population are poorly understood. We analysed data from a national probability sample survey of households, the Health Survey for England 2010. A total of 943 heterosexual couples living together, where at least one partner was aged between 16–44 years, were included. We used correlation coefficients to measure the strength of similarities between partners with respect to demographic characteristics, general health, health behaviours and sexual history. Males were on average 2 years older than their female partners, although this age difference ranged from a median of 0 years in men aged 16–24 years to a median of 2 years in men aged 35–44 years. A positive correlation between partners was found for all demographic characteristics. With respect to general health and health behaviours, a strongly positive correlation was found between men and women in reporting alcohol consumption at ⩾3 days a week and smoking. Men typically reported greater numbers of sexual partners than their female partner, although men and women with more partners were more likely to mix with each other. We have been able to elucidate the patterns of sexual mixing between men and women living together in England. Mixing based on demographic characteristics was more assortative than sexual characteristics. These data can better inform mathematical models of STI transmission.

## INTRODUCTION

Research on transmission dynamics of sexually transmitted infections (STIs) has frequently focused on the distribution of sexual behaviours (e.g. numbers of partners, concurrency and condom use) of individuals in the population in relation to demographic characteristics (e.g. age, ethnicity) [1–3], and their association with STI risk. Less attention has been given to the measurement of sexual mixing patterns and the behaviours of partners, which also influences both individual and population transmission risk [4].

Assortative mixing is said to occur when people have sex primarily with those with similar characteristics (e.g. sexual behaviours and demographic characteristics) [5] to themselves. If a population subgroup with a sufficient rate of partner change mixes little with those outside the group, i.e. assortative mixing, this will sustain undetected STI transmission within the group but with little spread to others outside (e.g. maintenance of HIV spread among men who have sex with men). Conversely, disassortative mixing occurs when people have sex with others from different lifestyle groups. This is associated with the spread of STIs through the whole population [6, 7]. Mixing between partners can be said to be random when it is neither assortative nor disassortative.

The purpose of mathematical models is to aid understanding of the dynamics of STI transmission and to guide appropriate interventions and predictions of their impacts [8]. Models that incorporate mixing matrices indicate that the degree of assortative mixing impacts STI transmission [5, 8]. However, empirical population data to inform models are scarce [9]. More generally, knowledge of the extent of assortative mixing in a general population is needed to guide sexual health policy. Where the degree of assortative mixing is high, STI prevention may be best focused on those at highest risk. In contrast, where mixing is largely disassortative, STIs may be more widely disseminated in the population and population screening may be more appropriate.

To date, many studies of sexual mixing have used convenience samples of STI clinic attendees [6, 10, 11]. These target high-risk populations and are thus not representative of the general population. Probability sample surveys have typically collected data on the characteristics of sexual partners as reported by individual study participants [4, 12], and are thus largely restricted to demographic rather than behavioural mixing patterns. However, the 2010 Health Survey for England (HSE 2010), a national probability sample survey, collected data from all adults within households, thus allowing collection of detailed information from both members of a live-in couple. For the first time in the survey's history, HSE 2010 included questions on key self-reported sexual behaviours. This provides an opportunity to compare the reporting of demographic characteristics and behaviours from individuals currently living as couples in England.

The objectives of this paper are to present and describe the mixing patterns by demographic characteristics, general health, health behaviours and sexual history, and to ascertain whether mixing differs from that expected through random partner selection.

## METHODS

### Data source

The 2010 Health Survey for England (HSE 2010) is an annual survey, monitoring the health of the population living in private households in England. Details of HSE 2010 methodology have previously been published [13]. Briefly, HSE 2010 uses a multi-stage stratified random probability sampling design, using addresses from the Small User Postcode Address File as the sampling frame. At each selected household, all adults aged >16 years, up to a total of 10, were invited to participate. A total of 8420 adults participated with an individual response rate of 59% [13], which is line with other major social surveys recently completed in Britain [14, 15]. For the first time in 2010, questions from the National Survey of Sexual Attitudes and Lifestyles (Natsal), Britain's national probability survey of sexual behaviour [16], were included in a sexual health module asked to participants aged 16–69 years.

### Participants

Inclusion in the analyses presented here is restricted to opposite-sex couples living together (i.e. reporting that they are in a married or cohabiting relationship with the other person responding to the survey), in which at least one partner was aged 16–44 years and both completed the sexual health module and reported ever being sexually active. For older partnerships, it becomes increasingly likely that at least one person might be older than the eligibility age for the sexual health module, thus excluding the partnership from analysis. The age restriction proposed ensures a better sample of partnerships not truncated by this age structure. Same-sex couples are excluded due to their small number and as patterns of mixing are recognized as being different to opposite-sex couples [17–19].

### Outcomes

HSE 2010 collected information on participant's demographic characteristics using face-to face interviews; general heath and health behaviours via face-to-face and self-completion pen-and-paper questionnaires; and sexual history by self-completion only. We compared couples' demographic characteristics in terms of age, ethnicity, socioeconomic class [defined as the three-class description of the National Statistics Socioeconomic classification (NS-SEC)] [20], and highest academic qualification. General health and health behaviours included frequency of alcohol consumption, current smoking status, body mass index (BMI) category [21], current mental illness [12-item General Health Questionnaire (GHQ-12)  score] [22], longstanding illnesses, and feelings of anxiety. Sexual history included consistency of condom use in the last 4 weeks, age at first sexual intercourse, numbers of opposite-sex partnerships (ever), numbers of opposite-sex partnerships (past year), having a STI diagnosis (ever), any same-sex experience (ever), and any same-sex experience (past 5 years).

### Statistical analysis

Due to small numbers of participants in ethnic minority groups, we combined these into: Asian (including Indian, Pakistani, Bangladeshi, other Asian background and mixed-white-Asian); Black (including Caribbean, African, other black background, mixed-white-black); and Other (Chinese, any other mixed background, any other background).

We present the observed prevalence of each characteristic by gender. Using these results we calculated the expected percentage for each possible combination of characteristics between men and women – the expected ‘mixing distribution’ if mixing is at random. As a descriptive measure we compared the expected mixing distribution with the observed mixing distribution.

To formally test the hypothesis of mixing at random we used a bivariate probit model for binary and ordinal outcomes. This model estimates a conditional correlation coefficient for the characteristic between partners after adjusting for the age and marital status of both partners. We tested whether the conditional correlation differs from zero, the value that represents mixing at random. A correlation coefficient >0 indicates assortative mixing as there is a greater association between partners' reported characteristics than expected by random, and conversely a coefficient <0 indicates disassortative mixing. As an indicator of strength of assortativity we refer to Newman's assortativity coefficient, which describes coefficients ⩾0·35 as assortative, 0·26–0·34 as moderately assortative and 0·15–0·25 as minimally assortative [23]. As a sensitivity analysis we produced correlation coefficients for the characteristics between partners stratified by marital status and categories of partnership age difference.

All statistical analyses were performed using the complex survey functions of Stata v. 12·1 (StataCorp LP, USA), taking into account the clustering, stratification and household weighting of the sample. The data were weighted to adjust for household selection probability and non-response bias by age/sex and region profile of the population of England [13]. Statistical significance was considered as *P* < 0·05 for all analyses.

## RESULTS

A total of 5454 participants in HSE 2010 were in live-in partnerships, of which 736 participants were excluded from this analysis as their partner did not take part in the survey. This left a sample of 4718 participants and thus a total of 2359 couples. Of these, 2347 were opposite-sex couples, which included, 1891 couples where both partners were eligible for the sexual health module. Of these, 943 couples included at least one participant aged 16–44 years and are analysed here.

### Demographic characteristics

**Fig. 1.**
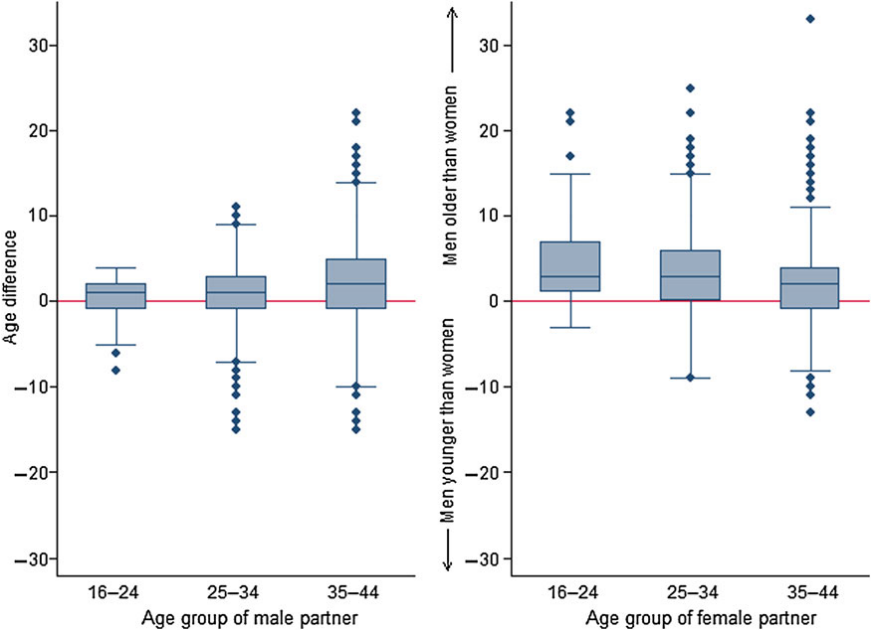
Distribution of age differences between partners, by age group and gender of each participant. Age difference calculated as female partner's age subtracted from male partner's age.

The median age of males and females was 37 [interquartile range (IQR) 31–42] years and 35 (IQR 29–40) years, respectively (Table 1). The unadjusted correlation of age within partnerships was highly assortative (rho = 0·96). The median age difference between partners was 2 years; however, this difference varied by age group. Male respondents aged 16–24 years at interview had a median difference of 0 (IQR −1 to 2) years with their partner. The median and spread of this difference increased with age to a median difference of 2 (IQR −1 to 5) years, such that on average, men aged 35–44 years were 2 years older than their partner (Fig. 1). The opposite relationship was observed for women. Women aged 16–24 years were on average 3 years younger than their male partner, a difference that reduced with increasing age (Fig. 1).

**Table 1.** Individual participant characteristics, by gender

	Men (percentage or median)	Women (percentage or median)
**Demographic characteristics**		
Age, years	37	35
Ethnicity		
White	84·9%	84·1%
Asian	8·3%	9·2%
Black	4·5%	3·7%
Other	2·2%	3·0%
National Statistics Socio-Economic Classification [20]		
Managerial and professional occupations	45·3%	42·4%
Intermediate occupations	19·7%	24·7%
Routine and manual occupations	35·0%	32·9%
Highest academic qualification		
No academic qualification	10·0%	7·9%
Academic qualification typically gained at age 16 years	27·6%	27·2%
Academic qualification typically gained at age 18 years	31·2%	30·5%
Degree qualification	31·2%	34·4%
**General health and health behaviours**		
Alcohol consumption		
Never	10·4%	18·0%
Twice a week or less	56·6%	61·7%
3 days a week or more	33·0%	20·3%
Smoking history		
Never	62·1%	62·1%
Ex-smoker	19·1%	19·1%
Current smoker	18·9%	18·9%
Body mass index		
Underweight	1·0%	1·9%
Normal	27·6%	48·0%
Overweight	45·3%	28·4%
Obese	26·1%	21·8%
Current mental illness (GHQ-12) [22]		
No illness	88·2%	85·9%
Has illness	11·8%	14·1%
Any longstanding illness		
No illness	72·0%	70·9%
Has illness	28·0%	29·1%
Longstanding mental illness		
No illness	97·7%	97·1%
Has illness	2·3%	2·9%
Feeling anxious/depressed at time of interview		
No	84·7%	79·2%
Moderate or extreme	15·3%	20·8%
**Sexual history**		
Always used a condom, past 4 weeks		
No	84·1%	84·0%
Yes	15·9%	16·0%
Age at first heterosexual intercourse, years	17	17
Number of heterosexual partners, lifetime		
1	14·6%	21·0%
2	8·0%	11·7%
3–4	17·5%	24·3%
5–9	27·7%	26·1%
⩾10	32·2%	16·9%
Number of heterosexual partners, past year		
0	1·1%	1·0%
1	97·5%	98·2%
⩾2	1·4%	0·8%
STI diagnosis, ever		
No	91·3%	84·3%
Yes	8·7%	15·7%
Same-sex experience, ever		
No	99·7%	97·1%
Yes	0·3%	2·9%
Same-sex experience, past 5 years		
No	100·0%	98·2%
Yes	0·0%	1·8%

GHQ-12, 12-item General Health Questionnaire; STI, Sexually transmitted infection.

We present the mixing distribution for ethnic groups of couples (Table 2). Given the observed distribution of ethnic groups among men and women (Table 1), under assumptions of random mixing, we would expect both partners to be white in 71% of couples. The observed percentage is 82%, giving an observed:expected ratio of 1·2. The greatest ratio of observed to expected percentage of couples reporting the same ethnicity was found in the Black ethnic group, the observed percentage was 16 times that expected by random mixing. The ratio was 10 for Asian ethnicity. We formally analysed each ethnicity in turn producing separate 2 × 2 tables of the combination of binary outcomes of belonging to that ethnicity or not (Table 3). We would expect matching by white/non-white ethnicity, where neither partner is white or both partners are white, in 74% of couples under random mixing (Table 3). We observed 95% matching and a positive conditional correlation between partners [rho = 0·96, 95% confidence interval (CI) 0·94–0·98]. Similar results were found for ethnicity defined by both Asian/non-Asian and Black/non-Black (rho = 0·99, 95% CI 0·98–1·00, and rho = 0·96, 95% CI 0·90–0·99, respectively). With respect to social class, matching responses between partners were greater than expected. The ratio of observed percentages relative to expected was greatest in the routine/manual occupations  (1·7), compared to managerial (1·4) and intermediate (1·3) occupations. An ordinal model illustrated a significant conditional correlation between social class of male and female partners (rho = 0·49, 95% CI 0·41–0·56). There was also an assortative relationship with having a degree qualification.

**Table 2.** Percentage observed (expected) of ethnicity mixing, for couples

Male ethnicity	Female ethnicity							
	White		Asian		Black		Other	
	Observed % (expected %)	O/E	Observed % (expected %)	O/E	Observed % (expected %)	O/E	Observed % (expected %)	O/E
White	81·5 (70·8)	1·2	1·4 (8·0)	0·2	0·7 (3·2)	0·2	1·0 (2·6)	0·4
Asian	0·5 (7·0)	0·1	7·9 (0·8)	9·9	0·0 (0·3)	0·0	0·0 (0·3)	0·0
Black	1·3 (4·0)	0·3	0·1 (0·5)	0·2	3·2 (0·2)	16·0	0·2 (0·1)	2·0
Other	0·3 (1·8)	0·2	0·0 (0·2)	0·0	0·0 (0·1)	0·0	1·9 (0·1)	19·0

O, Observed; E, expected.

**Table 3.** Observed and expected mixing distribution of demographic, general health and sexual history, for couples

	Observed	Expected	O/E	rho (95% CI)*	*P* value
**Demographic characteristics**					
Ethnicity: White					
Neither	13·0%	2·4%	5·4	0·96 (0·94 to 0·98)	<0·0001
Both	81·9%	71·3%	1·1		
Male only	3·0%	13·6%	0·2		
Female only	2·1%	12·7%	0·2		
Ethnicity: Asian					
Neither	90·3%	83·2%	1·1	0·99 (0·98 to 1·00)	<0·0001
Both	7·9%	0·8%	10·2		
Male only	0·5%	7·6%	0·1		
Female only	1·4%	8·5%	0·2		
Ethnicity: Black					
Neither	94·8%	91·9%	1·0	0·96 (0·90 to 0·99)	<0·0001
Both	3·0%	0·2%	17·8		
Male only	1·5%	4·4%	0·3		
Female only	0·7%	3·5%	0·2		
National Statistics Socio-Economic Classification [20]					
Both: managerial	27·4%	19·9%	1·4	0·49 (0·41 to 0·56)	<0·0001
Both: intermediate	6·2%	4·7%	1·3		
Both: routine/manual	19·1%	11·2%	1·7		
Males higher group	24·7%	33·2%	0·7		
Females higher group	22·6%	31·0%	0·7		
Has a degree qualification					
Neither	55·1%	45·1%	1·2	0·66 (0·57 to 0·73)	<0·0001
Both	20·7%	10·7%	1·9		
Male only	10·5%	20·5%	0·5		
Female only	13·7%	23·7%	0·6		
**General health and health behaviours**					
Frequent alcohol consumption (at least 3 days/week)					
Neither	62·8%	53·4%	1·2	0·74 (0·67 to 0·80)	<0·0001
Both	16·1%	6·7%	2·4		
Male only	16·9%	26·4%	0·6		
Female only	4·1%	13·6%	0·3		
Current smoker					
Neither	67·6%	59·7%	1·1	0·67 (0·59 to 0·74)	<0·0001
Both	12·9%	5·0%	2·6		
Male only	13·6%	21·4%	0·6		
Female only	6·0%	13·9%	0·4		
Body mass index					
Both: underweight	0·4%	0·0%	24·4	0·22 (0·13 to 0·31)	<0·0001
Both: normal	15·0%	12·8%	1·2		
Both: overweight	13·7%	13·0%	1·1		
Both: obese	8·7%	5·8%	1·5		
Men higher group	41·8%	43·8%	1·0		
Women higher group	20·5%	24·5%	0·8		
Current mental illness (GHQ-12)					
Neither	76·2%	75·6%	1·0	0·13 (-0·04 to 0·29)	0·1208
Both	2·3%	1·7%	1·4		
Male only	9·6%	10·2%	0·9		
Female only	11·9%	12·5%	0·9		
Any longstanding illness					
Neither	55·2%	51·1%	1·1	0·32 (0·20 to 0·43)	<0·0001
Both	12·3%	8·1%	1·5		
Male only	15·7%	19·9%	0·8		
Female only	16·8%	20·9%	0·8		
Longstanding mental illness					
Neither	95·7%	94·8%	1·0	0·74 (0·41 to 0·90)	<0·0001
Both	0·9%	0·1%	13·3		
Male only	1·4%	2·2%	0·6		
Female only	2·0%	2·9%	0·7		
Feeling anxious/depressed at time of interview					
Neither	68·3%	66·8%	1·0	0·20 (0·05 to 0·34)	0·0066
Both	4·8%	3·3%	1·5		
Male only	10·8%	12·3%	0·9		
Female only	16·1%	17·7%	0·9		
**Sexual history**					
Always used a condom, past 4 weeks					
Both: none	79·5%	70·8%	1·1	0·88 (0·81 to 0·93)	<0·0001
Both: yes	11·2%	2·5%	4·5		
Male only	5·1%	13·8%	0·4		
Female only	4·2%	12·9%	0·3		
Heterosexual sex before age 16 years					
Neither	69·7%	66·6%	1·0	0·36 (0·21 to 0·49)	<0·0001
Both	6·3%	3·3%	1·9		
Male only	15·3%	18·4%	0·8		
Female only	8·7%	11·8%	0·7		
Number of heterosexual partners, lifetime					
Both: 1	9·5%	3·1%	3·1	0·57 (0·51 to 0·63)	<0·0001
Both: 2	2·1%	1·0%	2·1		
Both: 3–4	4·7%	4·1%	1·1		
Both: 5–9	9·7%	7·2%	1·3		
Both: ⩾10	8·7%	5·4%	1·6		
Men higher group	45·8%	50·6%	0·9		
Women higher group	19·4%	28·7%	0·7		
Number of heterosexual partners, past year					
Both: 0	0·3%	0·0%	30·2	0·57 (0·23 to 0·79)	0·0001
Both: 1	96·0%	95·7%	1·0		
Both: ⩾2	0·1%	0·0%	7·6		
Men higher group	2·1%	2·4%	0·9		
Women higher group	1·6%	1·9%	0·8		
STI diagnosis, ever					
Neither	78·0%	76·9%	1·0	0·25 (0·05 to 0·43)	0·0115
Both	2·4%	1·4%	1·7		
Male only	6·3%	7·3%	0·9		
Female only	13·3%	14·4%	0·9		
Same-sex experience, ever					
Neither	96·7%	96·6%	1·0	0·48 (0·20 to 0·69)	0·0001
Both	0·1%	0·0%	8·7		
Male only	0·2%	0·4%	0·6		
Female only	3·0%	3·1%	1·0		

O, Observed; E, expected; GHQ-12, 12-item General Health Questionnaire; STI, Sexually transmitted infection.

* Conditional correlations between partner outcomes from a bivariate probit model adjusting for age and marital status.

### General health and health behaviours

Couples were assortative with respect to reporting alcohol consumption ⩾3 days a week, current smoking status, and reporting a longstanding mental illness (rho = 0·74, 95% CI 0·67–0·80; 0·66, 95% CI 0·57–0·73; and 0·74, 95% CI 0·41–0·90, respectively). Other general health markers (e.g. anxiety at time of interview, BMI, any longstanding illness) indicated moderate assortativeness, while data for the measure of current mental illness (GHQ-12) were consistent with random mixing.

### Sexual history

With respect to reporting having always used condoms in the last 4 weeks, we observed a greater prevalence of matching responses than expected 91% *vs*. 73%, respectively (Table 3), reflected by a greatly assortative conditional correlation of rho = 0·88 (95% CI 0·81–0·93). With respect to the reported number of opposite-sex partnerships in a lifetime, we again observed assortative mixing (rho = 0·57, 95% CI 0·50–0·63). A similar correlation was found considering numbers of opposite-sex partners in the past year. To a lesser extent, partners were also correlated with respect to reporting first heterosexual intercourse before age 16 years and same-sex experience (ever).

Further analysis revealed that the degree of assortativity in demographic characteristics, health and sexual history was largely similar whether or not couples were similar in age (Supplementary Table S1); and whether or not partners were married or cohabiting (Supplementary Table S2).

## DISCUSSION

There are high degrees of assortative mixing based on demographic, general health and sexual history for individuals living together as couples in England. Uniquely, using probability sample survey data collected from both individuals in the couple, we have also been able to describe the mixing distribution between sexual partners, which will aid the parameterization of future mathematical models of STI transmission.

Mixing based on age is strongly assortative, but we note the pattern of mixing also differs by gender and age, as others have reported [4, 19, 24]. Females tend to engage in sexual relationships with men older than themselves, especially when at a young age [24], while the age range of male partners is narrower among older women [4]. Such age differences, in part, explain the higher risk of STI acquisition in young females than young males [3], such that age mixing is included as a UNAIDS indicator of sexual risk behaviour [25]. Our results were also consistent with research suggesting strong assortative mixing by ethnicity which is thought to sustain the different STI epidemiologies in different ethnic groups [26–28].

The degree of assortativity regarding demographic characteristics was greater than for many variables corresponding to general health and sexual history. This finding may arise because information about an individual's sexual history, including their experience of STI diagnosis/diagnoses, is unlikely to be disclosed at first meeting. In contrast, demographic characteristics (e.g. ethnicity and age) may be guessed, therefore allowing this information to influence interactions with a potential future sexual partner [29]. These social factors may also reflect how or where partners meet (e.g. at an educational establishment or through the workplace).

With respect to the measure of health, assortative correlations were reported for alcohol consumption and smoking. As well as markers of health these also describe behaviour which, like the demographic characteristics, can be used to inform a person's decision as to partner selection. Longstanding mental illness was strongly assortative, possibly because a longer exposure to an individual with a longstanding mental illness may in turn influence one's own long-term mental state, or because of compatibility influencing partnership formation. However, in contrast, recent anxiety and current mental illness (GHQ-12) showed minimal or no assortativity, which may be more likely to be influenced by new distressing experiences rather than a partner's anxiety.

No difference was found in the degree of assortative mixing observed between married and non-married cohabiting couples indicating that mixing patterns may be similar, and justifies our decision to present results combined by marital status.

Mathematical models of STI transmission are greatly influenced by the degree of assortative mixing in relation to previous partner numbers [9]. We found that numbers of partners, both over the lifetime and in the past year, show assortative correlations. These were lower than for the demographic variables suggesting that partnerships are more disassortative with respect to partner numbers. Condom use in the last 4 weeks had a correlation greater than that of partner numbers, and similar to that observed for the demographic characteristics studied. Given the recent time-frame and the monogamous nature of nearly all cohabiting relationships, we would expect reports to tally.

In contrast to previous studies [4, 12, 26, 30], an important strength of our study is that we were able to examine data collected from both individuals in a couple rather than relying upon one participant to report about their own behaviour and that of their partner. Asking both partners should be more accurate, so our estimates of mixing are likely to be more reliable than previous studies, improving the parameterization of mathematical models of STI transmission. However, using data from a household survey means that our estimates are not necessarily generalizable to non-cohabiting partners. Previous research suggests that non-cohabiting partnerships display less assortative mixing than cohabiting sexual partnerships, especially casual partnerships [4]. Furthermore, casual partners in particular constitute a non-negligible proportion of all sexual partnerships [4], and are an important influence on STI transmission in the population because of their typically short duration and high partner change rate. Although, as a proportion of the population of sexually active people (rather than partners), the majority live with their sexual partner [31].

This study has also been able to examine a greater range of characteristics than has been examined before, including social class, alcohol consumption, mental illness, and sexual history. Despite this greater number of variables, HSE 2010 did not collect data on the length of the relationship. Partner similarity at interview may be due to convergence of characteristics over time facilitated by a shared environment, rather than assortative selection. However, research into partner convergence is inconclusive [32]. Moreover, an individual's characteristics at time of interview may be different to that at time of partnership formation.

As with all survey data, these are subject to reporting bias. In particular, although the data were collected using self-completion questionnaires from all eligible household members, the data may have been subject to some social desirability bias, especially if participants were concerned that others in their home may find out previously undisclosed sensitive information, such as having had a STI diagnosis.

A further strength of this study is the use of the bivariate probit model to assess the correlation between partners. Unlike the Newman assortativity coefficient, previously used to measure assortativity [17, 23, 30, 33, 34], this allows us to control for predictors of outcome characteristics simultaneously for both partners before calculating the correlation coefficient. However, this means we are unable to make detailed comparisons between the coefficients we observe and what has been seen elsewhere.

In conclusion, this study is important as it provides nationally representative estimates of the patterns of sexual mixing among couples living together in England, and will enable better mathematical models of STI transmission in the general population of England, although the results and methods will be of interest and applicable to other similar populations.

## SUPPLEMENTARY MATERIAL

For supplementary material accompanying this paper visit http://dx.doi.org/10.1017/S0950268814002155.

## References

[1] Fenton KA, Sexual behaviour in Britain: reported sexually transmitted infections and prevalent genital Chlamydia trachomatis infection. Lancet 2001; 358: 1851–1854. 11741624 10.1016/S0140-6736(01)06886-6

[2] Thomas JC, Tucker MJ The development and use of the concept of a sexually transmitted disease core. Journal of Infectious Diseases 1996; 174: S134–S143. 8843243 10.1093/infdis/174.supplement_2.s134

[3] Sonnenberg P, Prevalence, risk factors, and uptake of interventions for sexually transmitted infections in Britain: findings from the National Surveys of Sexual Attitudes and Lifestyles (Natsal). Lancet 2013; 382: 1795–1806. 24286785 10.1016/S0140-6736(13)61947-9 PMC3899025

[4] Mercer CH, Who has sex with whom? Characteristics of heterosexual partnerships reported in a national probability survey and implications for STI risk. International Journal of Epidemiolgy 2008; 38: 206–214. 10.1093/ije/dyn216 19001667

[5] Boily MC, Poulin R, Masse B Some methodological issues in the study of sexual networks: from model to data to model. Sexually Transmitted Diseases 2000; 27: 558–571. 11099071 10.1097/00007435-200011000-00004

[6] Aral SO, Sexual mixing patterns in the spread of gonococcal and chlamydial infections. American Journal of Public Health 1999; 89: 825–833. 10358670 10.2105/ajph.89.6.825 PMC1508665

[7] Anderson RM, Garnett GP Mathematical models of the transmission and control of sexually transmitted diseases. Sexually Transmitted Diseases 2000; 27: 636–643. 11099079 10.1097/00007435-200011000-00012

[8] Garnett GP An introduction to mathematical models in sexually transmitted disease epidemiology. Sexually Transmitted Infections 2002; 78: 7–12. 11872850 10.1136/sti.78.1.7 PMC1763694

[9] Turner KME, Investigating ethnic inequalities in the incidence of sexually transmitted infections: mathematical modelling study. Sexually Transmitted Infections 2004; 80: 379–385. 15459406 10.1136/sti.2003.007575 PMC1744908

[10] Barlow D, Daker-White G, Band B Assortative sexual mixing in a heterosexual clinic population – a limiting factor in HIV spread? AIDS (London, England) 1997; 11: 1039–1044. 10.1097/00002030-199708000-00013 9223739

[11] Garnett GP, Sexual mixing patterns of patients attending sexually transmitted diseases clinics. Sexually transmitted diseases 1996; 23: 248–257. 8724517 10.1097/00007435-199605000-00015

[12] Kenyon C, Colebunders R Birds of a feather: homophily and sexual network structure in sub-Saharan Africa. International Journal of STD & AIDS 2013; 24: 211–215. 23535354 10.1177/0956462412472455

[13] Craig R, Mindell J (eds). Health Survey for England 2010. Volume 1: Respiratory Health. Leeds: The NHS Information Centre, 2011

[14] Park A, (eds). British Social Attitudes: the 28th Report. London: NatCen Social Research, 2012

[15] Speight S, (eds). Childcare and Early Years Survey of Parents 2008. London: NatCen Social Research, 2009

[16] Erens B, National Survey of Sexual Attitudes and Lifestyles 3: Technical Report. 2013 (http://www.natsal.ac.uk/natsal-3/methodology). Accessed 11 December 2013

[17] Kurdek LA, Schmitt JP Partner homogamy in married, heterosexual cohabiting, gay, and lesbian couples. Journal of Sex Research 1987; 23: 212–232.

[18] Schwartz CR, Mare RD Trends in educational assortative marriage from 1940 to 2003. Demography 2005; 42: 621–646. 16463914 10.1353/dem.2005.0036

[19] Jepsen LK, Jepsen CA An empirical analysis of the matching patterns of same-sex and opposite-sex couples. Demography 2002; 39: 435–453. 12205751 10.1353/dem.2002.0027

[20] Office of National Statistics. Standard Occupational Classification 2010: *volumes 1–3*. Basingstoke: Palgrave Macmillan, 2010.

[21] World Health Organization. BMI classification. (http://apps.who.int/bmi/index.jsp?introPage=intro_3.html). Accessed 18 December 2013.

[22] Goldberg D, Williams P A User's Guide to the General Health Questionnaire. Slough: NFER-Nelson, 1988.

[23] Doherty IA, Schoenbach VJ, Adimora AA Sexual mixing patterns and heterosexual HIV transmission among African Americans in the Southeastern United States. Journal of Acquired Immune Deficiency Syndromes 2009; 52: 114–120. 19506485 10.1097/QAI.0b013e3181ab5e10 PMC2741169

[24] Gregson S, Sexual mixing patterns and sex-differentials in teenage exposure to HIV infection in rural Zimbabwe. Lancet 2002; 359: 1896–1903. 12057552 10.1016/S0140-6736(02)08780-9

[25] Slaymaker E A critique of international indicators of sexual risk behaviour. Sexually Transmitted Infections 2004; 80: ii13–21. 15572635 10.1136/sti.2004.011635 PMC1765852

[26] Laumann EO, Youm Y Racial/ethnic group differences in the prevalence of sexually transmitted diseases in the United States: a network explanation. Sexually Transmitted Diseases 1999; 26: 250–261. 10333277 10.1097/00007435-199905000-00003

[27] Fenton K, Johnson AM, Nicoll A Race, ethnicity, and sexual health. British Medical Journal 1997; 314: 1703. 9202498 10.1136/bmj.314.7096.1703 PMC2126878

[28] Aral SO Patterns of sexual mixing: mechanisms for or limits to the spread of STIs? Sexually Transmitted Infections 2000; 76: 415–416. 11221121 10.1136/sti.76.6.415 PMC1744256

[29] Kalmijn M Intermarriage and homogamy: causes, patterns, trends. Annual Review of Sociolology 1998; 24: 395–421. 10.1146/annurev.soc.24.1.395 12321971

[30] Bohl DD, McFarland W, Raymond HF Improved measures of racial mixing among men who have sex with men using Newman's assortativity coefficient. Sexually Transmitted Infections 2011; 87: 616–620. 21983853 10.1136/sextrans-2011-050103

[31] Mercer CH, Changes in sexual attitudes and lifestyles in Britain through the life course and over time: findings from the National Surveys of Sexual Attitudes and Lifestyles (Natsal). Lancet 2013; 382: 1781–1794. 24286784 10.1016/S0140-6736(13)62035-8 PMC3899021

[32] Humbad MN, Is spousal similarity for personality a matter of convergence or selection? Personality and Individual Differences 2010; 49: 827–830. 21116446 10.1016/j.paid.2010.07.010 PMC2992433

[33] Newman ME Assortative mixing in networks. Physical Review Letters 2002; 89: 28. 10.1103/PhysRevLett.89.208701 12443515

[34] Schwartz C, Graf N Assortative matching among same-sex and different-sex couples in the United States, 1990–2000. Demographic Research 2009; 21: 843–878. 20333322 10.4054/demres.2009.21.28 PMC2843104

