# Supplementary material for: Patterns of sexual mixing with respect to social, health and sexual characteristics among heterosexual couples in England: analyses of probability sample survey data
Source: Epidemiol Infect. 2014 Aug 28;143(7):1500–10. doi: 10.1017/S0950268814002155 (PMC4411639; doi:10.1017/S0950268814002155)
Supplement: Supplementary file 1 [file S0950268814002155sup001.doc]

**Supplementary Material**

Epidemiology and Infection

**Patterns of sexual mixing with respect to social, health and sexual characteristics among heterosexual couples in England: Analyses of probability sample survey data**

P. Prah, A.J. Copas, C.H. Mercer, A. Nardone, and A.M. Johnson

| Table S1: Conditional correlations between partner outcomes, by age difference (female partner’s age subtracted from male partner’s age) | | | |
| --- | --- | --- | --- |
|  | < less than 0 years | 0 – 5 years | greater than 5 years |
| **Demographic characteristics** |  |  |  |
| Ethnicity: White | 0.91 (0.76, 0.97) | 0.97 (0.93, 0.99) | 0.97 (0.91, 0.99) |
| Ethnicity: Asian | 1.00 (0.99, 1.00) | 0.99 (0.97, 1.00) | 1.00 (0.97, 1.00) |
| Ethnicity: Black | 0.89 (0.53, 0.98) | 0.97 (0.81, 0.99) | 1.00 (1.00, 1.00) |
| National Statistics Socio-Economic Classification[^20^](#_ENREF_20) | 0.49 (0.33, 0.62) | 0.48 (0.35, 0.58) | 0.51 (0.36, 0.64) |
| Has a degree qualification | 0.71 (0.55, 0.82) | 0.67 (0.55, 0.76) | 0.53 (0.31, 0.7) |
|  |  |  |  |
| **General health and health behaviour** |  |  |  |
| Frequent drinking (at least 3 days/week) | 0.73 (0.56, 0.84) | 0.77 (0.68, 0.84) | 0.74 (0.56, 0.85) |
| Current smoker | 0.60 (0.4, 0.75) | 0.72 (0.6, 0.81) | 0.66 (0.46, 0.8) |
| Body mass index | 0.35 (0.18, 0.49) | 0.23 (0.11, 0.36) | 0.15 (-0.07, 0.36) |
| Current mental illness (GHQ-12)[^22^](#_ENREF_22) | 0.15 (-0.19, 0.46) | 0.16 (-0.08, 0.37) | 0.08 (-0.29, 0.43) |
| Any longstanding illness | 0.35 (0.14, 0.54) | 0.38 (0.21, 0.52) | 0.25 (0.01, 0.47) |
| Longstanding mental illness | 0.59 (-0.04, 0.88) | 0.80 (0.35, 0.95) | 0.57 (0.04, 0.85) |
| Feeling anxious/depressed at time of interview | 0.30 (-0.01, 0.56) | 0.26 (0.05, 0.45) | 0.02 (-0.27, 0.31) |
|  |  |  |  |
| **Sexual history** |  |  |  |
| Always used a condom, past 4 weeks | 0.90 (0.73, 0.97) | 0.87 (0.76, 0.93) | 0.94 (0.8, 0.99) |
| Heterosexual sex before age 16 years | 0.34 (0.03, 0.59) | 0.35 (0.13, 0.54) | 0.42 (0.06, 0.68) |
| Number of heterosexual partners, lifetime | 0.52 (0.36, 0.66) | 0.56 (0.47, 0.64) | 0.69 (0.55, 0.79) |
| Number of heterosexual partners, past year | 0.04 (-0.06, 0.13) | 0.72 (0.27, 0.91) | N/A |
| STI diagnosis, ever | 0.36 (-0.07, 0.68) | 0.3 (0.02, 0.54) | 0.09 (-0.29, 0.45) |
| Same-sex experience, ever | N/A | N/A | N/A |
| Correlations are calculated using a bivariate probit model adjusting for marital status | | |  |
| N/A - omitted due to small sample size | |  |  |

| Table S2: Conditional correlations between partner outcomes, by marital status | | |
| --- | --- | --- |
|  | Married | Cohabiting |
| **Demographic characteristics** |  |  |
| Ethnicity: White | 0.98 (0.96, 0.99) | 0.78 (0.54, 0.9) |
| Ethnicity: Asian | 1.00 (0.99, 1.00) | -0.73 (-0.79, -0.65) |
| Ethnicity: Black | 0.97 (0.9, 0.99) | 0.90 (0.67, 0.97) |
| National Statistics Socio-Economic Classification[^20^](#_ENREF_20) | 0.52 (0.43, 0.61) | 0.40 (0.25, 0.54) |
| Has a degree qualification | 0.65 (0.54, 0.74) | 0.66 (0.49, 0.78) |
|  |  |  |
| **General health and health behaviours** |  |  |
| Frequent drinking (at least 3 days/week) | 0.77 (0.69, 0.84) | 0.67 (0.51, 0.79) |
| Current smoker | 0.60 (0.47, 0.71) | 0.75 (0.63, 0.84) |
| Body mass index | 0.23 (0.11, 0.34) | 0.21 (0.05, 0.37) |
| Current mental illness (GHQ-12)[^22^](#_ENREF_22) | 0.18 (-0.04, 0.38) | 0.07 (-0.22, 0.34) |
| Any longstanding illness | 0.28 (0.14, 0.41) | 0.41 (0.2, 0.58) |
| Longstanding mental illness | 0.82 (0.45, 0.95) | 0.53 (0.09, 0.79) |
| Feeling anxious/depressed at time of interview | 0.14 (-0.05, 0.31) | 0.34 (0.08, 0.56) |
|  |  |  |
| **Sexual history** |  |  |
| Always used a condom, past 4 weeks | 0.92 (0.86, 0.96) | 0.78 (0.56, 0.90) |
| Heterosexual sex before age 16 years | 0.29 (0.08, 0.47) | 0.42 (0.18, 0.61) |
| Number of heterosexual partners, lifetime | 0.61 (0.53, 0.68) | 0.48 (0.34, 0.6) |
| Number of heterosexual partners, past year | 0.60 (0.17, 0.84) | 0.58 (-0.03, 0.87) |
| STI diagnosis, ever | 0.36 (0.12, 0.56) | 0.00 (-0.36, 0.37) |
| Same-sex experience, ever | N/A | N/A |
| Correlations are calculated using a bivariate probit model adjusting for age | | |
| N/A - omitted due to small sample size | |  |
